# A light-induced nitric oxide controllable release nano-platform based on diketopyrrolopyrrole derivatives for pH-responsive photodynamic/photothermal synergistic cancer therapy†
†Electronic supplementary information (ESI) available. See DOI: 10.1039/c8sc03386b


**DOI:** 10.1039/c8sc03386b

**Published:** 2018-08-29

**Authors:** Ya Wang, Xiaoyu Huang, Yunyun Tang, Jianhua Zou, Peng Wang, Yewei Zhang, Weili Si, Wei Huang, Xiaochen Dong

**Affiliations:** a Key Laboratory of Flexible Electronics (KLOFE) , Institute of Advanced Materials (IAM) , Nanjing Tech University (NanjingTech) , 30 South Puzhu Road , Nanjing 211800 , China . Email: iamxcdong@njtech.edu.cn ; Email: iamwlsi@njtech.edu.cn; b Shaanxi Institute of Flexible Electronics (SIFE) , Northwestern Polytechnical University (NPU) , 127 West Youyi Road , Xi'an 710072 , China; c Department of Hepatobiliary and Pancreatic Surgery , Zhongda Hospital , Medical School , Southeast University , Nanjing 210009 , China . Email: zhangyewei@njmu.edu.cn

## Abstract

Emerging treatment approaches, such as gas therapy (GT), photodynamic therapy (PDT) and photothermal therapy (PTT), have received widespread attention. The development of an intelligent multifunctional nano-platform responding to tumor microenvironments for multimodal therapy is highly desirable. Herein, a near-infrared (NIR) light-responsive nitric oxide (NO) photodonor (4-nitro-3-trifluoromethylaniline, NF) and a pH-sensitive group (dimethylaminophenyl) have been introduced into a diketopyrrolopyrrole core (denoted as DPP-NF). The DPP-NF nanoparticles (NPs) can be activated under weakly acidic conditions of lysosomes (pH 4.5–5.0) to generate reactive oxygen species (ROS) and enhance photothermal efficiency. The fluorescence detection demonstrated that NO controllable release can be realized by “on–off” switching of the NF unit under NIR light irradiation or dark conditions. The controllable NO release of DPP-NF NPs can not only trigger tumor cell death by DNA damage, but also overcome PDT inefficiencies caused by hypoxia in tumors. Additionally, DPP-NF NPs displayed 45.6% photothermal conversion efficiency, making them superior to other reported DPP derivatives. *In vitro* studies showed that DPP-NF NPs possessed low dark toxicity and high phototoxicity with a half-maximal inhibitory concentration of about 38 μg mL^–1^. *In vivo* phototherapy indicated that DPP-NF NPs exhibited excellent tumor phototherapeutic efficacy with passive targeting of the tumor site *via* the enhanced permeability and retention (EPR) effect. These results highlight that the nano-platform has promising potential for NO-mediated multimodal synergistic phototherapy in clinical settings.

## Abstract 2

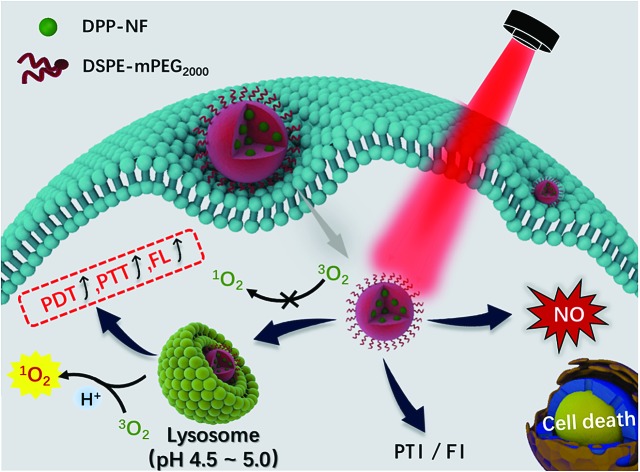
An intelligent multifunctional nano-platform responsive to the tumor microenvironment was established, which showed NO controllable “on–off” release and enhanced photodynamic/photothermal synergistic cancer therapy.

## Introduction

Despite the rapid development of diagnosis and treatment processes, cancer remains the biggest threat to human life and health in the world today. Traditional cancer treatments, such as chemotherapy,^1^–^3^ radiotherapy,^4^ and surgery, have some inevitable disadvantages. Drug resistance, significant side effects, lack of targeting, and normal cell damage further reduce the survival rate of patients. Photodynamic therapy (PDT) and photothermal therapy (PTT) have now been considered as novel alternatives to traditional oncology treatments with high selectivity, low toxicity, no drug resistance and lower non-invasiveness.^5^–^13^ PDT and PTT depend on photon energy absorbed by photosensitizers to generate reactive oxygen species (ROS) or heat, causing damage or death of blood vessels and tumor cells.^14^–^18^ Owing to the difference of pH value between the tumor microenvironment (pH 4.5–5.0) and normal tissues (pH 7.4),^19^–^22^ smart pH-activated photosensitizers are extremely desirable. Lysosomes with weak acidity in tumour cells can act as a pH-activated reactor,^23^–^25^ which provides the most effective approach for imaging and therapy *in vitro* and *in vivo*.

As a star molecule, NO is ubiquitous in organisms and is involved in the regulation of blood multicellular activities, including vascular growth, smooth muscle relaxation, immune response, apoptosis, and synaptic transmission. In addition to its role in normal physiological activities, a large number of studies have confirmed that it is closely related to the occurrence and development of many diseases, especially tumours.^26^–^29^ The biological effect of NO is a double-edged sword with concentration dependence. Generally, continuous low concentrations (pM to nM) of NO have a tumour promoting effect on the growth of vascular tumours.^30^–^32^ However, a high concentration of NO (>mM) may cause NO poisoning in blood. When NO is maintained at a certain concentration (nM to mM), it could suppress tumour growth and display an anti-tumour effect. The main mechanisms of the NO anti-tumour effect are as follows: (1) affecting the energy metabolism of cells and causing tumour cell death owing to energy metabolism disorders. (2) Binding with superoxide anions in cells and generating nitrogen/oxygen free radicals to damage DNA. (3) Mediating macrophage activation against tumours and inhibiting tumour metastasis by restraining platelet aggregation. (4) Inducing the apoptosis of tumour cells by activating the p53 expression.

As a gaseous free radical species biosynthesized in living cells, NO is a short-lived (<1 s) pleiotropic molecule with a small diffusion radius (<200 μm). Therefore, stimulus-responsive NO release at the tumour site becomes the key to realize cancer gas therapy (GT). In an attempt to deliver NO, Liu's group reported a multifunctional nano-platform N-GQDs@Ru–NO@Gal^33^ by using nitrogen-doped graphene quantum dots (N-GQDs) as a carrier and Ru–NO as a nitric oxide donor. Yang's group developed nitric oxide donor *S*-nitrosothiol-modified hollow bilayer polymer nanoparticles^32^ with pH and glucose dual responsiveness. However, some problems in NO therapy, such as low loading and fast release, still limit its application in cancer treatment. Besides, the metabolic process and safety assessment of NO carriers are also worthy of further study.

**Scheme 1:**
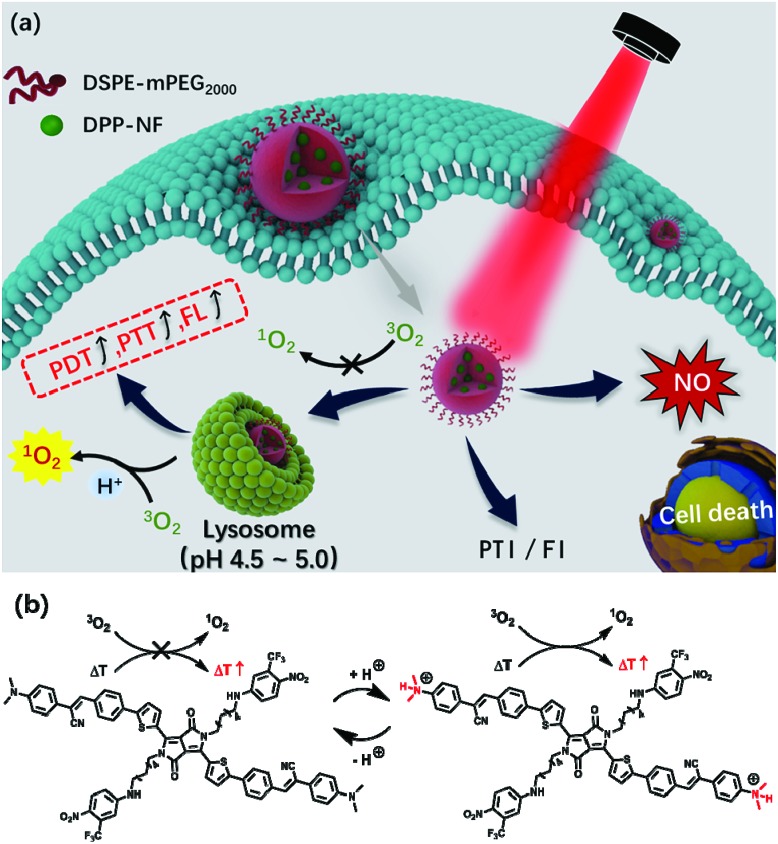
(a) Schematic illustration of pH-responsive DPP-NF NPs for PTI and FI guided PDT/PTT/GT synergistic cancer therapy. (b) Structural change of pH-responsive DPP-NF for enhanced singlet oxygen generation and photothermal effects.

Diketopyrrolopyrrole (DPP) derivatives^34^–^36^ as efficient photosensitizers have been widely explored for cancer phototherapy for their near-infrared (NIR) absorption, and high photo and thermal stability.^37^ Herein, a NIR light-responsive nitric oxide (NO) photodonor (4-nitro-3-trifluoromethylaniline, NF) and pH-sensitive unit (dimethylaminophenyl-) were covalently bonded to the diketopyrrolopyrrole core (denoted as DPP-NF). The resultant NIR DPP derivative can serve as both a pH-sensitive photosensitizer and NO delivery carrier, providing a possible approach to avoid the introduction of additional NO carriers. DPP-NF nanoparticles (NPs) could be prepared through reprecipitation, which solved the water solubility and biocompatibility of photosensitizers in physiological environments. More importantly, DPP-NF NPs could realize controllable “on–off” release of NO under light/dark conditions. Fluorescence imaging (FI) and photothermal imaging (PTI) demonstrated that DPP-NF NPs could be efficiently accumulated at the tumour site (Scheme 1a). As reported,^38^ the pH-sensitive characteristic of the photosensitizer came from the photoinduced electron transfer (PET) mechanism. Here, –NMe_2_ as the pH sensitive unit can be protonated under weakly acidic conditions. Under neutral conditions, the HOMO level of –NMe_2_ is between the HOMO and LUMO levels of the photosensitizer. After excitation, an electron transfers from –NMe_2_ to the HOMO of the photosensitizer, and the excited electrons tend to shunt the HOMO of –NMe_2_ rather than perform radiative relaxation and vibrational relaxation.^39^ Under acidic conditions, the addition of H^+^ to –NMe_2_ makes its HOMO lower than that of the photosensitizer, consequently activating the photosensitizer to the “on” state for photothermal and photodynamic effects^23^ (Scheme 1b). The DPP-NF NPs could be activated under a weakly acidic environment of lysosomes (pH 4.5–5.0) to enhance singlet oxygen generation and photothermal effects. Both *in vitro* and *in vivo* experiments suggested that DPP-NF NPs presented excellent antitumor efficiency by PDT, PTT and GT under laser irradiation.

## Results and discussion

### Characterization of DPP-NF NPs

**Scheme 2:**
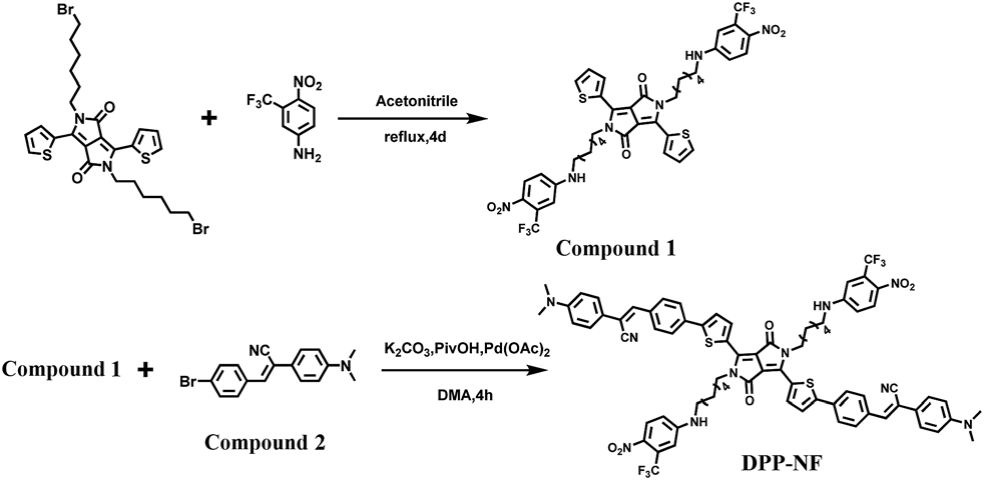
Synthetic routes of DPP-NF.

**Fig. 1:**
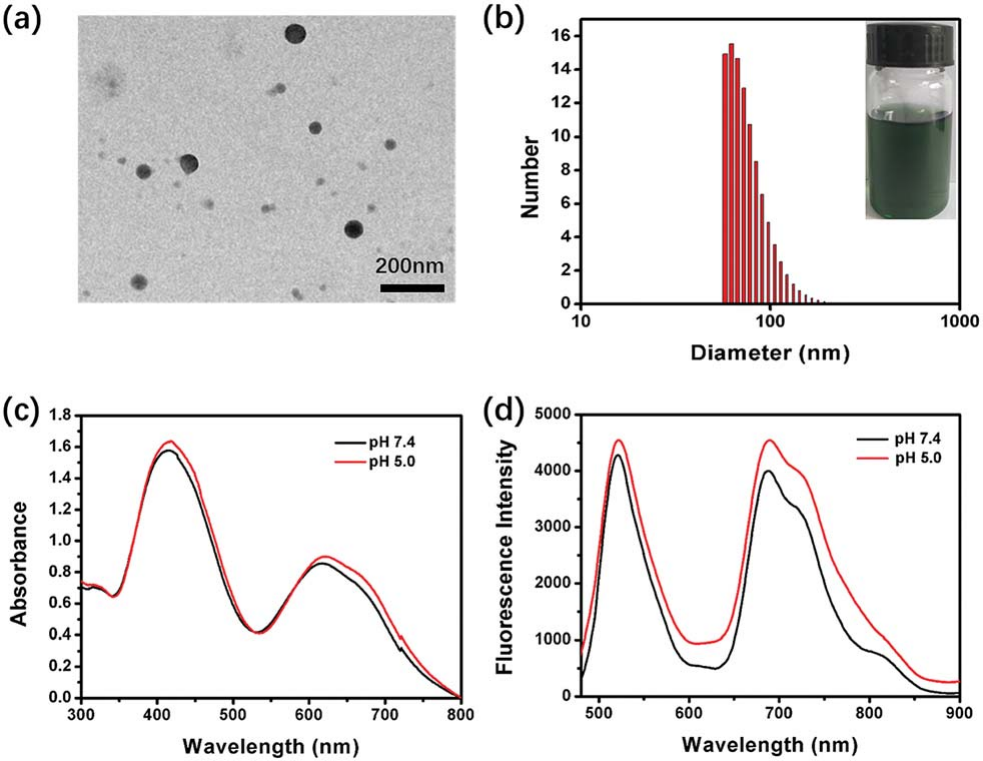
(a and b) TEM image and DLS of DPP-NF NPs (inset shows the DPP-NF NP aqueous solution). (c and d) UV-visible absorption and fluorescence emission spectra of DPP-NF NPs at different pH values (*λ*_ex_ = 480 nm).

Scheme 2 shows the synthetic route of DPP-NF. Nano-precipitation^40^ was adopted to prepare biocompatible DPP-NF NPs. Under vigorous stirring, the DPP-NF solution in tetrahydrofuran (5.0 mg mL^–1^) was dripped into a 1,2-distearoyl-*sn-glycero*-3-phosphoethanolamine–*N*-[methoxy(poly(ethylene glycol))-2000] (DSPE–mPEG_2000_) aqueous solution (0.1 mg mL^–1^), and then tetrahydrofuran was blown away with nitrogen to obtain a transparent green solution. After 30 days, the DPP-NF NP solution was still a clear aqueous solution without precipitation. A TEM image indicated that the obtained DPP-NF NPs presented a uniform spherical morphology with a size of around 60 nm (Fig. 1a). Dynamic light scattering (DLS) showed that the average hydrodynamic diameter was about 60–70 nm (Fig. 1b), which made it well passively target into the tumor site *via* the enhanced permeability and retention (EPR) effect. Due to the intermolecular π–π stacking^41^ between the aggregated DPP-NF molecules, the absorption of DPP-NF NPs exhibited a red shift and the absorption peak broadened compared to DPP-NF molecules in dichloromethane. As shown in Fig. 1c, DPP-NF NPs had strong absorption peaks at 418 nm and 623 nm, which were the characteristic absorption peaks of the nitric oxide photodonor and photosensitizer, respectively. What's more, the absorption peak at pH 5.0 was stronger than that at pH 7.4, indicating DPP-NF NPs could absorb more photon energy under acidic conditions. The fluorescence emission spectra in Fig. 1d show that DPP-NF NPs had high emission peaks at 521 nm and 692 nm, respectively. And the fluorescence intensity was significantly enhanced at pH 5.0, further demonstrating the pH-sensitive fluorescence characteristics of DPP-NF NPs.

### pH-activated photodynamic and photothermal performances

**Fig. 2:**
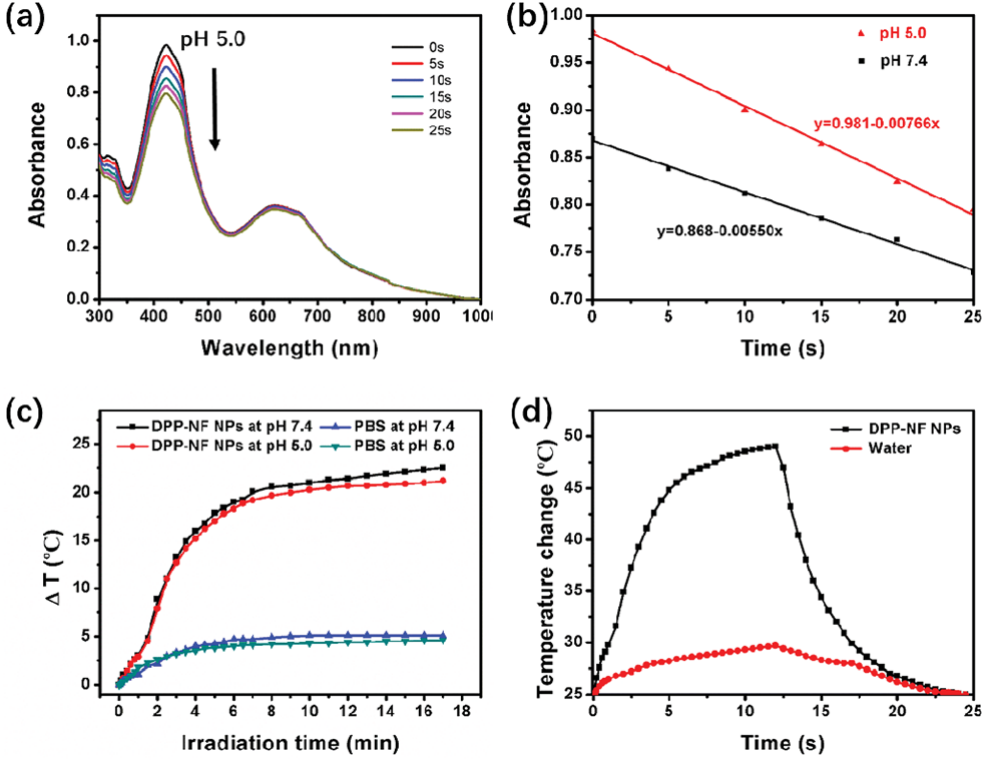
(a) Absorption spectra of DPP-NF NPs and DPBF for different irradiation times at pH 5.0. (b) Absorbance values of DPP-NF NPs and DPBF at pH 7.4 and pH 5.0 decreased with the increasing irradiation time. (c) Photothermal profiles of DPP-NF NP and PBS solutions irradiated with 660 nm lasers at different pH. (d) The photothermal effect of DPP-NF NP aqueous solution and water irradiated with a 660 nm laser.

To verify the activation of DPP-NF NPs under weakly acidic conditions, their singlet oxygen generation and photothermal performance was detected at different pH values (pH 7.4 and 5.0). 1,3-Diphenylisobenzofuran (DPBF)^42^ was used as the singlet oxygen fluorescence probe, and the mixed solution of DPP-NF NPs and DPBF was irradiated with a 660 nm laser (0.35 W cm^–2^). A UV-vis spectrophotometer was used to observe the degradation of DPBF absorbance at 414 nm along with the irradiation time. As shown in Fig. 2a, b and S1,† the change of absorption intensity indicated that more singlet oxygen was produced by DPP-NF NPs under acidic conditions. Simultaneously, the photothermal performance of DPP-NF NPs was measured using a 660 nm laser (0.8 W cm^–2^). As shown in Fig. 2c, at pH 5.0, the temperature of the DPP-NF NP solution increased by 22.5 °C after 17 minutes of laser irradiation, which was 1.3 °C higher than that at pH 7.4. Furthermore, no temperature decrease was observed after five cycles of the reversible heating and natural cooling, indicating its excellent thermal stability under light conditions (Fig. S2a†). Taking water as a reference, the temperature changes were measured (Fig. 2d and S2b†) and the photothermal conversion efficiency^43^ of DPP-NF NPs (60 μg mL^–1^) was calculated to be as high as 45.6%.

### Light-induced NO release

**Fig. 3:**
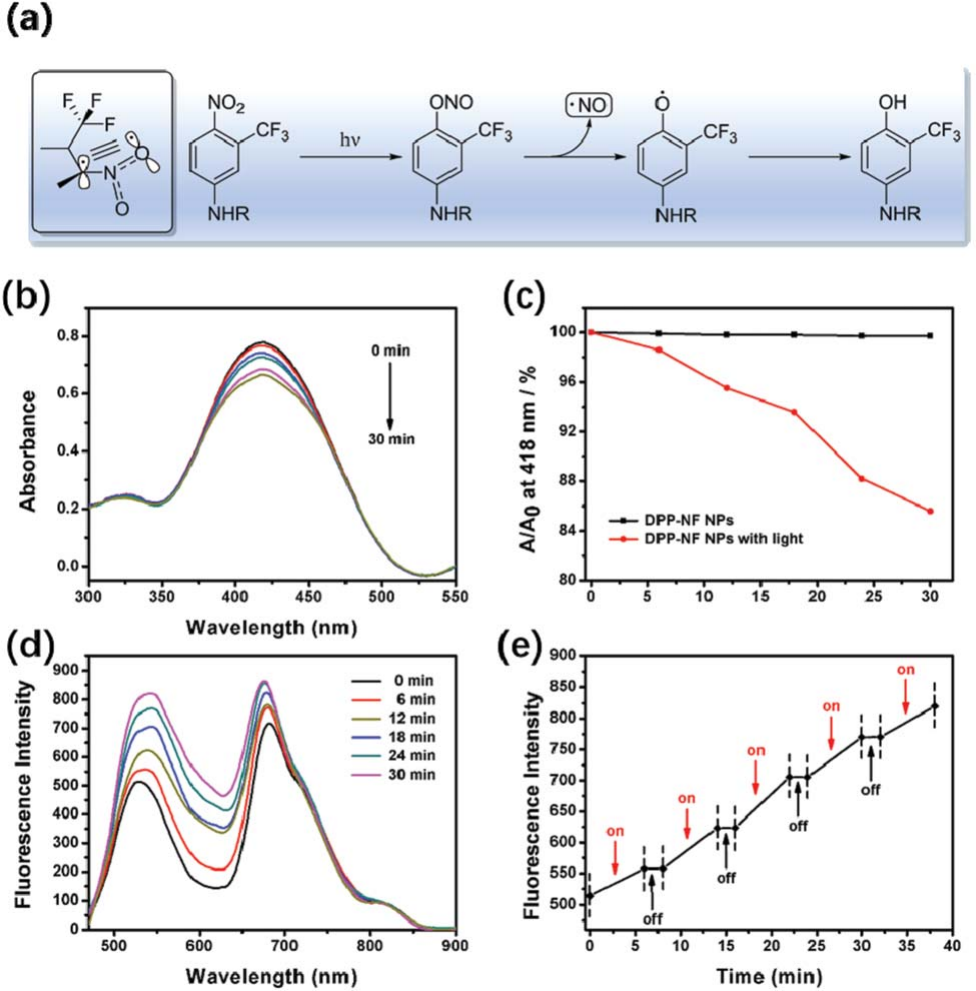
(a) The possible mechanism of nitric oxide photo-release. (b) The absorption spectra of the NO photodonor for different periods under 660 nm laser irradiation. (c) Comparison of the NO photodonor at the absorption peak of 418 nm under irradiation and dark conditions. (d) Fluorescence spectra of DPP-NF NPs and the DAF-FM DA mixture under 660 nm laser irradiation. (e) NO “on–off” controllable release curve obtained from (d).

Fig. 3a shows the NO release mechanism of the –NF unit. It can be observed that the nitro substituent of –NF is almost perpendicular to the plane of the aromatic ring due to the steric hindrance effect of –CF_3_. In the ground state and the excited state of the non-planar configuration, the p orbital of the oxygen atom overlaps with the adjoining aromatic ring. Under light irradiation, an optical rearrangement of the nitro–nitrite occurred and generated phenoxy and NO radicals.^44^,^45^ The light-induced absorption spectrum changes of DPP-NF NPs are shown in Fig. 3b and c. As shown in Fig. 3b, there was an absorption peak of the NO photodonor at 418 nm in the absorption spectrum. With the irradiation time increasing, the absorption intensity at 418 nm decreased. In contrast, no obvious decrease was observed in the absence of light irradiation (Fig. 3c). This phenomenon may come from the photo-bleaching of the NO photodonor, which is accompanied by the release of NO. To further verify the NO generation of the photodonor under the light irradiation, a NO fluorescent probe, 3-amino,4-aminomethyl-2′,7′-difluorescein diacetate (DAF-FM DA, 5 μM),^46^ was used to qualitatively detect the NO released from the DPP-NF NPs. As shown in Fig. 3d, with the irradiation time increasing, the intensity of the emission peak at 520 nm continuously enhanced due to the combination of the released NO with the probe. Additionally, fluorescence detection was used to confirm the controlled release of NO. As shown in Fig. 3e, the fluorescence intensity gradually increased after the irradiation. But, there is no obvious increase of the fluorescence intensity without light irradiation, demonstrating that the NO release of DPP-NF NPs could be controlled by NIR light irradiation. In addition, NO itself is extremely unstable and easily oxidized in cells or in the aqueous solution to generate NO_2_^–^. Under acidic conditions, NO_2_^–^ reacts with diazonium sulphonamides to produce diazo compounds, which in turn undergo coupling reactions with naphthalene vinyl diamines. The concentration of the product is linearly related to the concentration of NO^2–^, which exhibits a maximum absorbance at 540 nm. Therefore, the Griess assay^47^ was employed to identify the generation of NO by the DPP-NF NPs. A standard curve of this assay was established using NaNO_2_ (the product of NO and the Griess agent) first at known concentrations, and NO generation was calculated to be 66.2 μM per 100 μg mL^–1^ DPP-NF NP solution from the standard curve (Fig. S3†). These results suggested that DPP-NF NPs could be used as light-controlled NO delivering molecules to achieve gas therapy effects.

### *In vitro* experiment

**Fig. 4:**
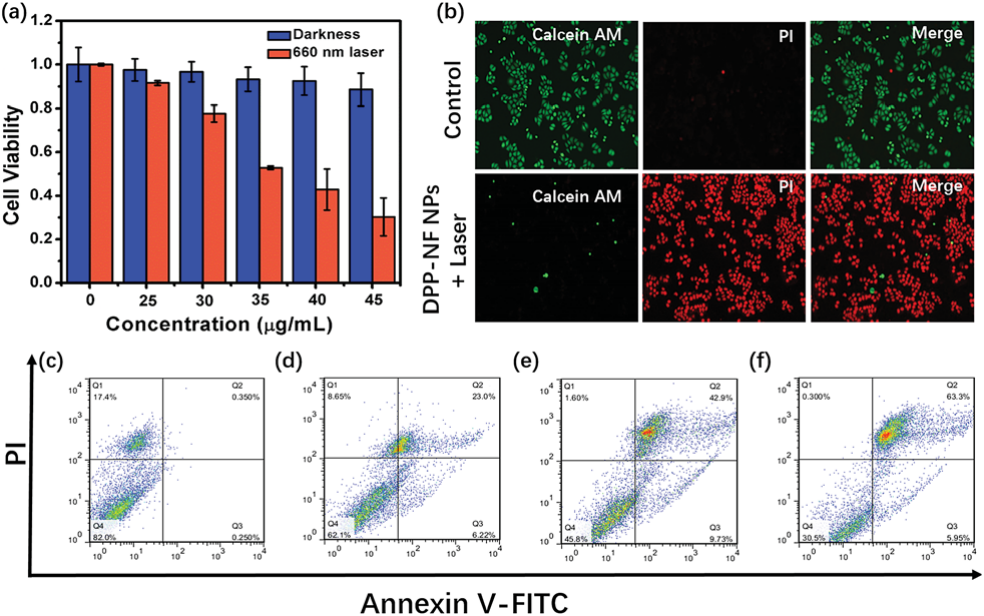
(a) Cell viability of HeLa cells treated with different concentrations of DPP-NF NPs measured by MTT (660 nm, 0.8 W cm^–2^). (b) Fluorescence images of HeLa cells co-stained with Calcein AM (live cells, green) and PI (dead cells, red) on the addition of DPP-NF NPs under dark and light conditions (660 nm, 0.8 W cm^–2^). Apoptosis of HeLa cells measured by flow cytometry: (c) blank, and DPP-NF NPs with laser irradiation at concentrations of (d) 30 μg mL^–1^, (e) 40 μg mL^–1^, and (f) 50 μg mL^–1^, respectively.

To explore the phototoxicity and dark toxicity of DPP-NF NPs, 3-(4,5-dimethyl-2-thiazolyl)-2,5-diphenyl-2-*H*-tetrazolium bromide (MTT) assay was performed on HeLa cells. As shown in Fig. 4a, the cell survival rate was significantly reduced with the concentration increase of DPP-NF NPs and its half-maximal inhibitory concentration (IC_50_) was about 38 μg mL^–1^, while the viability of the cells in the absence of irradiation remained at a relatively high level even at a high concentration of DPP-NF NPs. The low dark toxicity and high phototoxicity make DPP-NF NPs an excellent photosensitizer for cancer therapy. Besides, live–dead cell co-staining assays were also used to examine the cytotoxicity of DPP-NF NPs. Calcein-AM and pyridine iodide (PI) solutions can stain live and dead cells, respectively.^48^ Calcein-AM itself has no fluorescence; after reacting it with esterase in living cells to remove the AM group, the generated calcein can emit strong green fluorescence under the excitation of a 490 nm laser. PI can only pass through the dead cell membrane to reach the nucleus and embed the DNA and further generate red fluorescence under 535 nm laser excitation. As shown in Fig. 4b, the cell survival rate was obviously higher in the control group. In contrast, the cells appeared to die in large quantities with the addition of DPP-NF NPs and irradiation, which further proved the high phototoxicity and low dark toxicity of DPP-NF NPs.

Flow cytometric analysis could verify the therapeutic effect of DPP-NF NPs on tumour cells.^49^Fig. 4c shows that the cell survival rate was about 82% under dark conditions. When the concentration of DPP-NF increased to 30 μg mL^–1^, the early and late apoptosis of cells was 6.22% and 23.0%, respectively, with a cell viability of 62.1% (Fig. 4d). Similarly, when the DPP-NF NP concentration was 40 μg mL^–1^ (close to the IC_50_ value), its phototoxicity was significantly enhanced with a 45.8% cell survival rate and 42.9% late apoptosis rate (Fig. 4e). With the continuous increase of the DPP-NF NP concentration (50 μg mL^–1^), the cell late apoptosis increased to 63.3% and the survival rate was only 30.5% (Fig. 4f). It can be clearly observed that the proportion of cell late apoptosis increased, while the survival rate decreased and the proportion of early apoptosis was kept at a low level. Therefore, it can be concluded that DPP-NF NPs have an excellent tumour treatment effect as a photosensitizer.

**Fig. 5:**
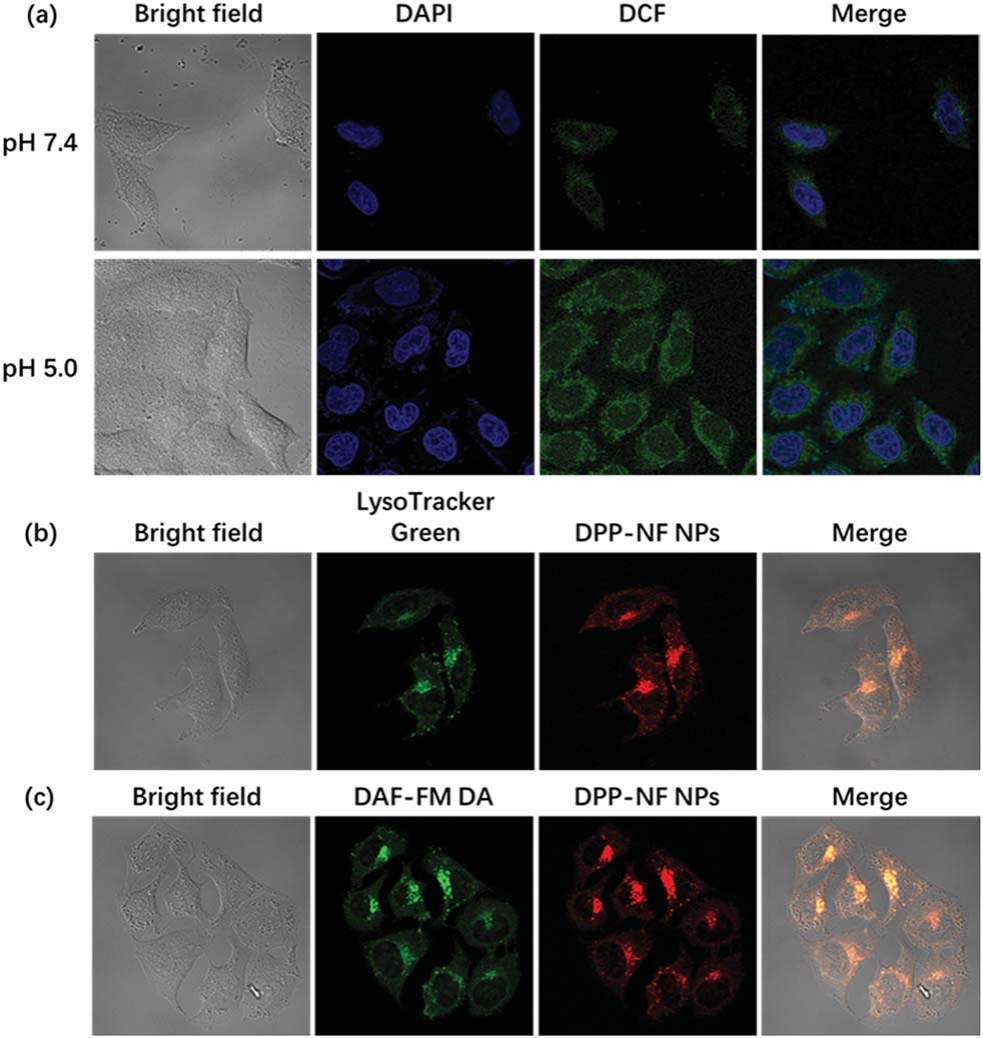
(a) Laser confocal images of DAPI and DCFH-DA stained HeLa cells pre-incubated with DPP-NF NPs at pH 7.4 and pH 5.0. (b) Laser confocal images of LysoTracker Green stained HeLa cells pre-incubated with DPP-NF NPs at pH 7.4. (c) Laser confocal images of DAF-FM DA stained HeLa cells pre-incubated with DPP-NF NPs at pH 7.4.

Compared with the cytoplasm of the neutral environment (pH 7.2), the weakly acidic environment of the lysosomes in the cells (pH 4.5–5.0) can activate the pH-sensitive photosensitizer. Under a confocal laser microscope, it could be observed that HeLa cells incubated at pH 5.0 had a significantly enhanced fluorescence intensity compared to the cells at pH 7.4 (Fig. S4a†). At the same time, using DCFH-DA^50^ as a ROS detection probe, in the control experiment, HeLa cells incubated with DPP-NF NPs and DCFH-DA without laser irradiation didn't cause any green fluorescence, indicating no ROS generation (Fig. S4b†). However, Fig. 5a shows that under laser irradiation, only weak green fluorescence was generated in HeLa cells at pH 7.4. When the pH value increased to 5.0, the green fluorescence intensity in the cells was significantly enhanced. Subcellular localization (staining with lysosomal green fluorescent probes) indicated that DPP-NF NPs were selectively localized in lysosomes (Fig. 5b). To verify that the NO photodonor can effectively produce NO in the cells in the presence of light irradiation, DAF-FM DA (5 μM), which can penetrate the cell membrane and be catalyzed by intracellular esterase to form DAF-FM, was used as the NO probe. DAF-FM itself has almost no fluorescence, but it can produce strongly green fluorescence after reacting with nitric oxide, evidenced in Fig. 5c. These observations further prove that DPP-NF NPs activated in lysosomes can greatly enhance the fluorescence intensity and ROS generation, which has a great effect on imaging and cell death.

### Dual-modal *in vivo* imaging

**Fig. 6:**
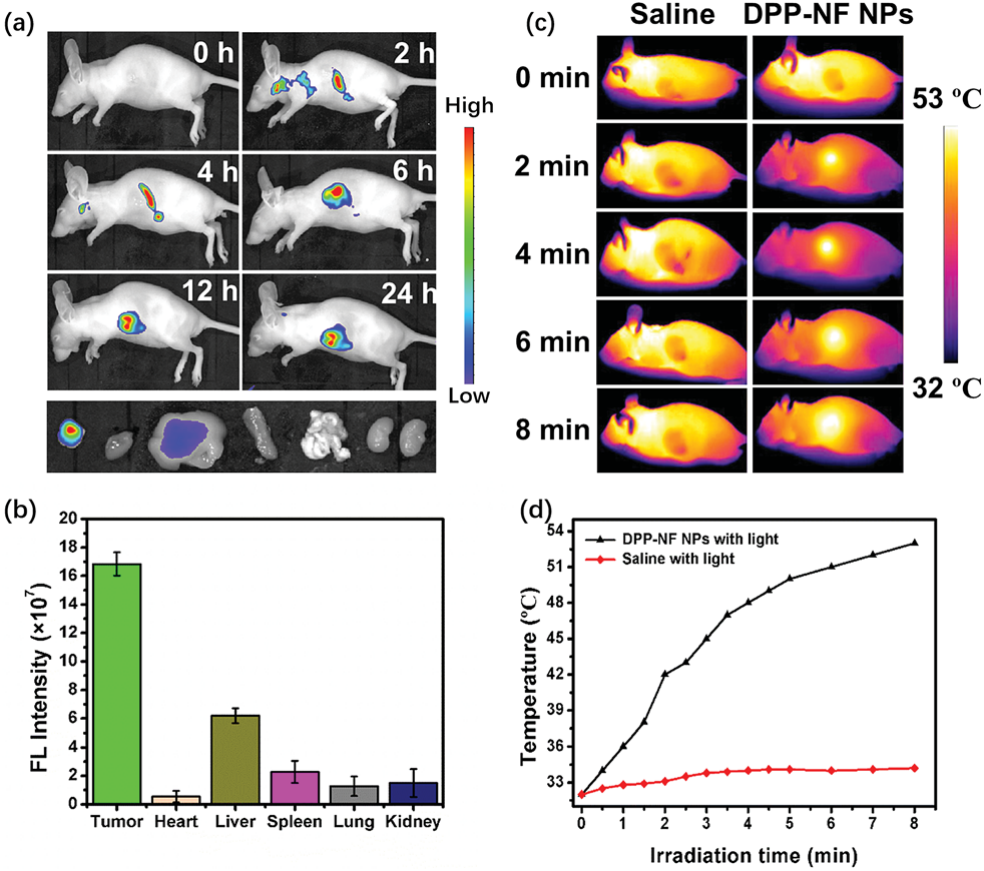
(a) Fluorescence imaging of tumour-bearing mice after intravenous injection of DPP-NF NPs. (b) Fluorescence intensity of the heart, liver, spleen, lung, and kidney of the dissected mice. (c) *In vivo* photothermal imaging of the tumour-bearing mice after intravenous injection of saline and DPP-NF NPs (100 μg mL^–1^) under laser irradiation. (d) The temperature curve of the tumour under laser irradiation (660 nm, 0.8 W cm^–2^).

Real-time imaging plays a vital role in cancer therapy and fluorescence imaging and can be used to monitor the accumulation and metabolism of DPP-NF NPs *in vivo*. As shown in Fig. 6a, no fluorescence appeared in mice before injection of the DPP-NF NP solution. As time went by, weak fluorescence of DPP-NF NPs could be observed around the tumour site after 2 h of intravenous injection. With DPP-NF NPs gradually enriched in the tumour site, the fluorescence intensity of DPP-NF NPs was observed to be maximal at about 6 h, indicating that DPP-NF NPs can be effectively targeted to the tumour by the EPR effect for phototherapy. Afterwards, the fluorescence intensity in mice gradually decreased, suggesting that DPP-NF NPs began to metabolize *in vivo*.^51^ From the fluorescence intensity of the heart, liver, spleen, lung, kidney and tumour of the dissected mice (Fig. 6c), it can be seen that DPP-NF NPs were mainly concentrated on the tumour site after injection for 24 h. And weak fluorescence in the liver demonstrated that DPP-NF NPs were primarily metabolized from the liver of mice. Due to the high photothermal conversion efficiency of DPP-NF NPs, photothermal imaging (PTI)^52^ could also be performed. After injection for 6 h (Fig. 6b), no significant temperature change was observed in the saline group under laser irradiation. However, the temperature of the tumour site increased obviously in the treatment group injected with DPP-NF NPs under irradiation (0.8 W cm^–2^). Within 8 minutes, the tumour temperature increased from 32 °C to 53 °C (Fig. 6d), which was sufficient to kill the tumour cells. In brief, both photothermal imaging and fluorescence imaging provide precise guidance for phototherapy of tumours.

### *In vivo* synergistic phototherapy

**Fig. 7:**
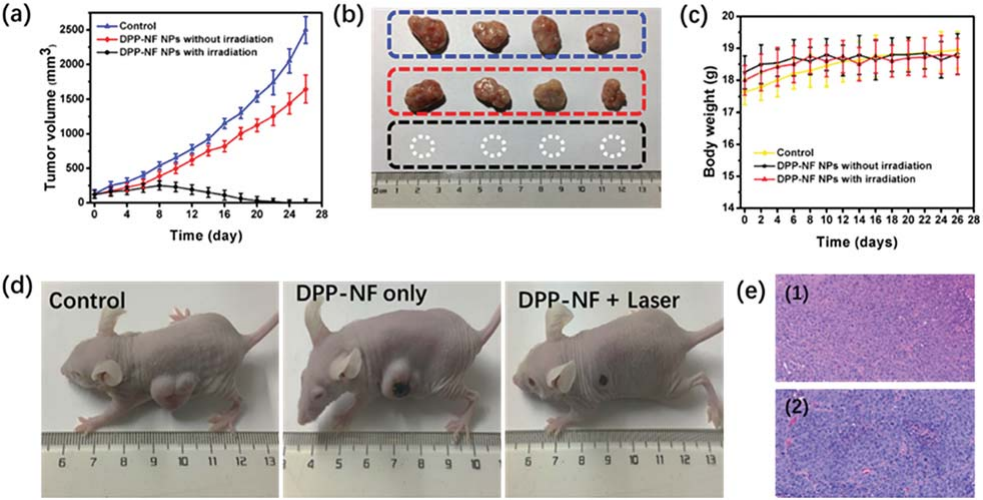
(a) Tumour growth curves of the mice in different groups. (b) Photographs of tumours from the mice in different groups treated with saline (top), DPP-NF NPs (middle), DPP-NF NPs and irradiation (bottom). (c) Changes in the body weight of the mice in different groups during treatment. (d) Photographs of the mice in different groups after 26 day treatment. (e) Histological microscopy images of the tumour after 26 day treatment: (1) control and (2) DPP-NF NPs.

To test the photodynamic, photothermal and NO therapeutic effects of DPP-NF NPs *in vivo*, HeLa-bearing mice were divided into three groups (4 mice each group): (i) the control group injected with saline only; (ii) the group injected with DPP-NF NPs without irradiation; (iii) the treatment group injected with DPP-NF NPs under laser exposure. Tumour volume changes were recorded to evaluate the phototherapy efficacy of DPP-NF NPs. During the treatment periods, the tumour volume of the mice in groups (i) and (ii) gradually increased, while the tumour volume in group (iii) decreased significantly from the eighth day after treatment (Fig. 7a). And the tumour of the mice almost completely disappeared after 12 days of treatment (Fig. 7b). In order to further confirm the therapeutic effect of DPP-NF NPs, the mice were fed for another two weeks, and no tumour re-occurrence was observed. Meanwhile, the survival rates of the HeLa tumor-bearing mice of the three groups are almost 100%. The weight of the mice of the three groups did not decrease significantly,^53^ (Fig. 7c) and photographs of the mice after 26 day treatment are also shown in Fig. 7d, demonstrating that DPP-NF NPs displayed excellent PDT, PTT and GT effects.

The cell-level therapeutic effect was also evaluated by H&E staining of the major organs (heart, liver, spleen, lungs, and kidney)^54^ for the three groups. As shown in Fig. 7e, the group injected with saline only and DPP-NF NPs only showed a good nuclear state of the tumour cells. H&E staining of the major organs (heart, liver, spleen, lungs, and kidney) of the three mice groups can help us arrive at a conclusion upon the cell-level therapeutic effect. Compared with the control group, the other two groups injected with DPP-NF NPs with irradiation or without irradiation showed no significant damage and inflammation of the major organs (Fig. S4†), further confirming that the DPP-NF NPs exhibited low side effects to normal tissues and good biocompatibility *in vivo*.

## Conclusion

In summary, a pH-sensitive diketopyrrolopyrrole derivative photosensitizer has been designed and synthesized with two dimethylaminophenyl units as a proton acceptor and –NF units as a NO photodonor. Through the reprecipitation approach, biocompatible DPP-NF NPs could be obtained. The pH-sensitive performance made DPP-NF NPs more active in weak acidic lysosomes over a neutral environment of cytoplasm, which could enhance singlet oxygen and photothermal effects greatly. By monitoring the light/dark process, DPP-NF NPs can achieve NO controllable release to solve the problem of excessive release. The dual modal imaging provided precise localization of the tumour site and prevented unexpected tissue damage. Additionally, both *in vitro* and *in vivo* studies demonstrated that DPP-NF NPs presented excellent tumour killing ability by NO-mediated and pH-activated photodynamic and photothermal synergetic therapy. The intelligent nano-platform based on DPP-NF NPs would be a promising approach for cancer phototherapy.

## Conflicts of interest

There are no conflicts to declare.

## Supplementary Material

Supplementary information
