# Supplementary material for: A light-induced nitric oxide controllable release nano-platform based on diketopyrrolopyrrole derivatives for pH-responsive photodynamic/photothermal synergistic cancer therapy
Source: Chem Sci. 2018 Aug 29;9(42):8103–9. doi: 10.1039/c8sc03386b (PMC6238752; doi:10.1039/c8sc03386b)
Supplement: Supplementary file 1 [file SC-009-C8SC03386B-s001.pdf]

Electronic Supplementary Material (ESI) for Chemical Science.
This journal is © The Royal Society of Chemistry 2018

Light-Induced Nitric Oxide Controllable Release Nano-platform Based on Diketopyrrolopyrrole Derivatives for pH-Responsive Photodynamic/Photothermal Synergistic Cancer Therapy

Ya Wang,[a] Xiaoyu Huang,[a] Yunyun Tang,[a] Jianhua Zou,[a] Peng Wang,[a] Yewei Zhang,[c] Weili Si,[a*] Wei Huang,[b] Xiaochen Dong[a*]*

[a]Key Laboratory of Flexible Electronics (KLOFE) & Institute of Advanced Materials (IAM), Nanjing Tech University (NanjingTech), 30 South Puzhu Road, Nanjing 211800, China.

E-mail: iamxcdong@njtech.edu.cn; iamwlsi@njtech.edu.cn

[b]Shaanxi Institute of Flexible Electronics (SIFE), Northwestern Polytechnical University (NPU), 127 West Youyi Road, Xi'an 710072, China.

[c]Department of Hepatobiliary and Pancreatic Surgery Zhongda Hospital, Medical School, Southeast University, Nanjing 210009, China.

E-mail: zhangyewei@njmu.edu.cn

1. Materials and Apparatus

1,6-Dibromohexane, Palladium acetate, Potassium Carbonate, 4-dimethylaminobenzaldehyde, 4-nitro-3-trifluoromethylaniline, 4-bromophenylacetonitrile, potassium hydroxide, pivalic acid, Dimethylacetamide (DMA), acetonitrile, ethanol and Sodium Sulfate were purchased from Admas. ^{1}H NMR and ^{13}C NMR spectra were recorded on Bruker DRX NMR spectrometer with $CDCl_3$ and tetramethylsilane (TMS) as internal standard. The absorption spectra were measured on UV-3600 UV-vis spectrophotometer (Shimadzu, Japan). The cells fluorescence imaging was recorded by confocal laser scanning microscope (Olympus IX 70 inverted microscope) and inverted fluorescence microscope (Nikon ECLIPSE Ts2R). Photothermal images are gained by an E_{50} infrared camera (FLIR, Arlington, VA).

2. Experimental section

2.1 Synthesis of compound 1

2,5-bis-(6-bromo-hexyl)-3,6-di-thiophen-2-yl-2,5-dihydro-pyrrolo[3,4-c]pyrrole-1,4-dione (3.130 g, 5 mmol), 4-nitro-3-trifluoromethylaniline (2.577 g, 12.5 mmol) and K_2CO_3 (1.725 g, 12.5mmol) were refluxed in acetonitrile for four days. The solvent was concentrated under reduced pressure, purified by colomn choromatograpy (EA/PE = 1:3, v/v). The product is red solid (1.971 g, yield: 45%). ^{1}H NMR (400 MHz, $CDCl_3$) δ 8.03 (m, 2H), 7.54 (d, J = 2.6 Hz, 2H),7.43 (d, J = 3.8 Hz, 4H), 7.07 (s, 2H), 6.81 (m, 2H), 4.05 (d, J = 4.5 Hz, 4H), 3.18 (d, J = 4.1 Hz, 4H), 1.68 – 1.59 (m, 16H). ^{13}C NMR (75 MHz, $CDCl_3$) δ 160.66, 153.41, 134.87, 133.10, 130.01, 128.90, 111.51, 108.99, 42.58, 41.71, 40.80, 40.25, 39.97, 39.69, 39.41, 31.45, 29.50, 28.23, 26.20.

2.2 Synthesis of compound 2

A mixed solution of 4-bromophenylacetonitrile (0.392 g) and 4-dimethylaminobenzaldehyde (0.298 g) in ethanol (50 mL) and KOH were stirred at 60 ºC for 24 h. Then the solvent was removed and purified by colomn choromatograpy (DCM/PE = 1:1, v/v) to produce a yellow solid (0.528 g, 81 % yield). ^{1}H NMR (300 MHz, $CDCl_3$) δ 7.87 (d, J = 8.8 Hz, 2H), 7.60 – 7.46 (m, 4H), 7.40 (s, 1H), 6.74 (d, J = 8.9 Hz, 2H), 3.08 (s, 6H). ^{13}C NMR (75 MHz, $CDCl_3$) δ 143.37, 131.97, 129.40, 126.84, 126.03, 118.61, 112.44, 99.99, 77.44, 77.02, 76.60, 67.33, 29.60, 25.56, 22.88, 13.94.

2.3 Synthesis of DPP-NF

The **compound 1** (1.752 g, 2 mmol), **compound 2** (1.630 g, 5 mmol), pivalic acid (0.061 g, 0.6 mmol), K_2CO_3 (0.69 g, 5 mmol), $Pd(OAc)_2$ (0.023 g, 0.10 mmol) were stirred in DMA (2 mL) at 110 ºC for 4 h under nitrogen atmosphere. After cooling to room temperature, the mixed solution was poured into 100 mL of saturated brine and extracted with DCM to remove DMA, then dried over anhydrous $NaSO_4$. The solvent was removed by rotary evaporation, which were then purified by colomn choromatograpy (DCM/Methanol = 100:1, v/v) to give DPP-NF as a green solid (yield 39 %). ^{1}H NMR (500 MHz, $CDCl_3$) δ 8.95 (m, 2H), 8.91 (m, 2H), 8.03 (d, J = 9.0 Hz, 2H), 7.68 (dd, J = 9.5, 4.9 Hz, 8H), 7.35 – 7.26 (m, 8H), 6.94 (s, 2H), 6.85 (s, 2H), 6.68 (d, J = 9.1 Hz, 2H), 3.43 (t, J = 6.6 Hz, 4H), 3.25 (t, J = 5.8 Hz, 4H), 3.02 (s, 12H) 1.90 (dd, J = 13.9, 6.9 Hz, 4H), 1.81 – 1.67 (m, 12H). ^{13}C NMR (75 MHz, $CDCl_3$) δ 151.82, 142.77, 134.60, 131.92, 131.40, 126.87, 121.78, 121.27, 119.04, 111.58, 104.54, 77.42, 77.00, 76.57, 46.01, 44.68, 42.98, 42.04, 40.73, 39.97, 36.52, 31.70, 31.03, 27.30, 24.27.

3. NO release assay

Griess assay can be used to determine the NO produced by the nano-platform. Different concentrations of $NaNO_2$ standard solution (2, 5, 10, 20, 40, 60, and 80 μM) were prepared. Each well of a 96-well plate was added with 100 μL of $NaNO_2$ standard solution. Then each well was added 50 μL Griess reagent 1 and Griess reagent 2, incubated each for 10 minutes and measured the absorbance of each well at 540 nm on a microplate reader. The relationship between the absorbance and the concentration of NO was build up by drawing a standard curve. According to the standard curve, we can get the NO concentration of the sample.

4. Lysosomal Location Study

Hela cells inoculated into glass bottom petri dish and cultured at 37 ºC, 5% (v/v) CO_2 for 24 h, media containing DPP-NF NPs (40 μg/mL, 2 mL) continued to incubate for another 24 h. After the media was aspirated, it was washed with PBS (pH 7.4) and 500 μL lysosomal green fluorescent probe was added and incubated at 37 ºC for 30 min. The probe was removed and the PBS solution was washed three times again, and finally 2 mL PBS solution was added. The fluorescence imaging of the cells was observed using confocal laser scanning microscope (Olympus IX 70 inverted microscope). DPP-NF NPs were excited at a wavelength of 633 nm and lysosomal green fluorescent probes were excited at 488 nm.

5. Live-dead cell staining experiments

Hela cells inoculated into glass bottom petri dish were incubated in media containing DPP-NF NPs (80 μg/mL, 2 mL) at 37 ºC under 5% (v/v) CO_2 for 24 h. The cells were irradiated

with a 660 nm laser (0.8 W/cm^2) for 8 min and stained after 6 h. The media in the petri dish was removed, washed with PBS three times, and then 1 mL of pyridine iodide (PI) and Calcein-AM staining solutions were added. The cells placed in a 37 ºC incubator for 40 minutes, after that the staining solution was aspirated and washed with PBS solution. Finally, the glass bottom petri dish was filled with 2 mL of PBS solution at pH 7.4. The cells staining results were obtained using an inverted fluorescence microscope (Nikon ECLIPSE Ts2R). Excited with 490 nm light, green living cells were observed while red dead cells were observed with 545 nm light excitation.

6. Flow cytometer assay

Hela cells seeded in six-well plates and divided into four groups, then cultured at 37 ºC under 5% (v/v) CO_2 for 24 h. The four wells were set as blank group, low concentration group, IC_{50} group, and high concentration group. After treating with different concentration of DPP-NF NPs for 24 h, the six-well plates was irradiated using a 660 nm laser (0.8 W/cm^2). The six-well plate was further incubated for 12 hours, the media was aspirated, then 200 μL of pancreatin and 1 mL of media was added to stop the digestion. Afterwards, the cells were collected, centrifuged for 5 minutes using a 1.5 mL centrifuge tube, the supernatant was aspirated, and washed twice with 500 μL of cold PBS. Finally, 500 μL binding buffer and 1 μL Fitc was added, 30 min later, adding 1 μL PI to flow tube. The signal was collected by a BD FACSCalibur flow cytometer (PerkinElmer) with 10^5 cells counted in each sample.

7. *In vivo* fluorescent imaging

Tumor-bearing mice were injected intravenously with DPP-NF NPs (100 μg/mL) and their

fluorescence was monitored at 0, 2, 4, 6, 12, and 24 h, respectively. After 24 h, the mice were sacrificed for heart, liver, spleen, lungs, kidney and tumor. Lastly, all fluorescence images were recorded by IVIS Lumina K Vivo Imaging System (PerkinElmer, U.S.A.).

8. *In vivo* combination therapy

Hela tumor-bearing nude mice were divided into three groups (4 mice per group) to study the anti-tumor effect of DPP-NF NPs. Nude mice in the control group were injected with saline, while the other two groups were injected with the same concentration of DPP-NF NPs (100 μg/mL). 6 h after the injection, the control group and treatment group were irradiated with a 660 nm laser for 8 minutes. The mouse body weight and tumor volume were recorded once in two days. At the end of the experiment, all mice were sacrificed. The tumors and major organs (heart, liver, spleen, lungs and kidney) were dissected, washed with saline, and placed in a 10% formalin solution for H&E staining.

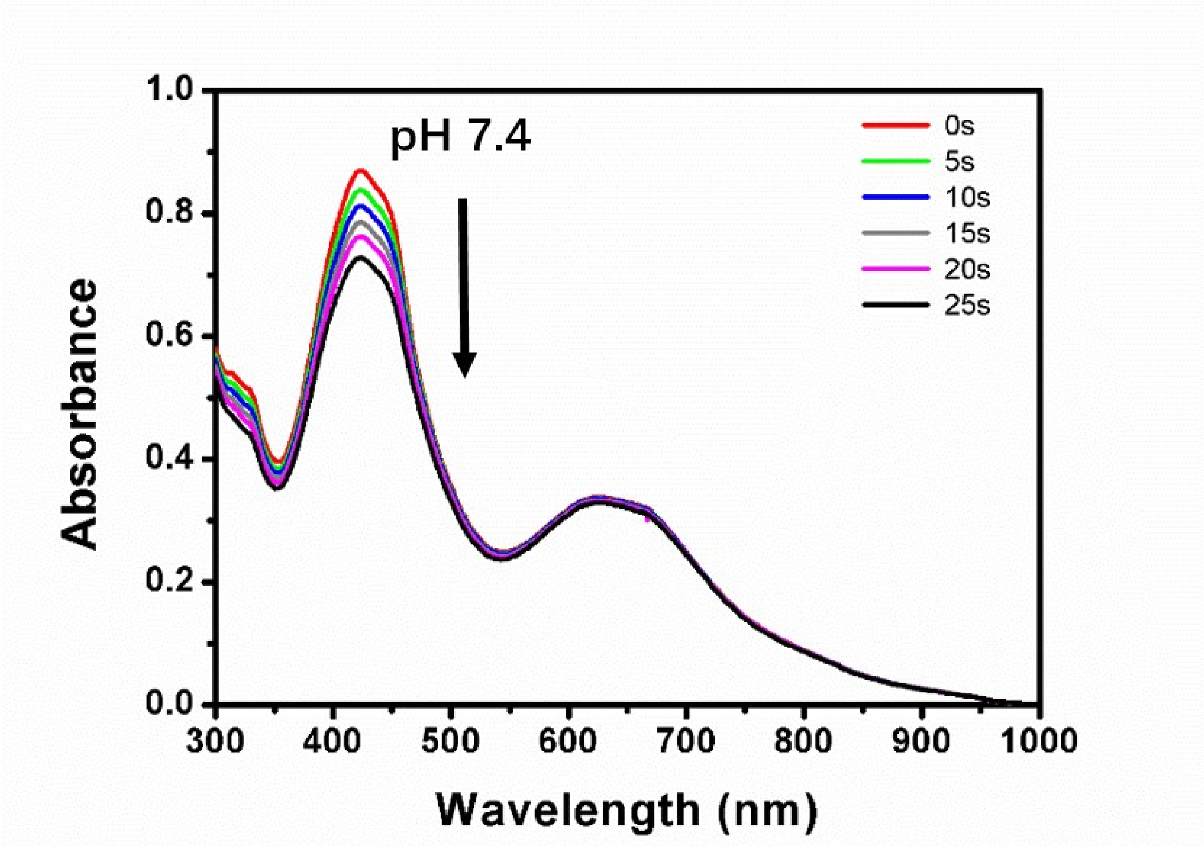

Figure S1 Absorption spectra of DPP-NF NPs and DPBF under different irradiation times (pH 7.4).

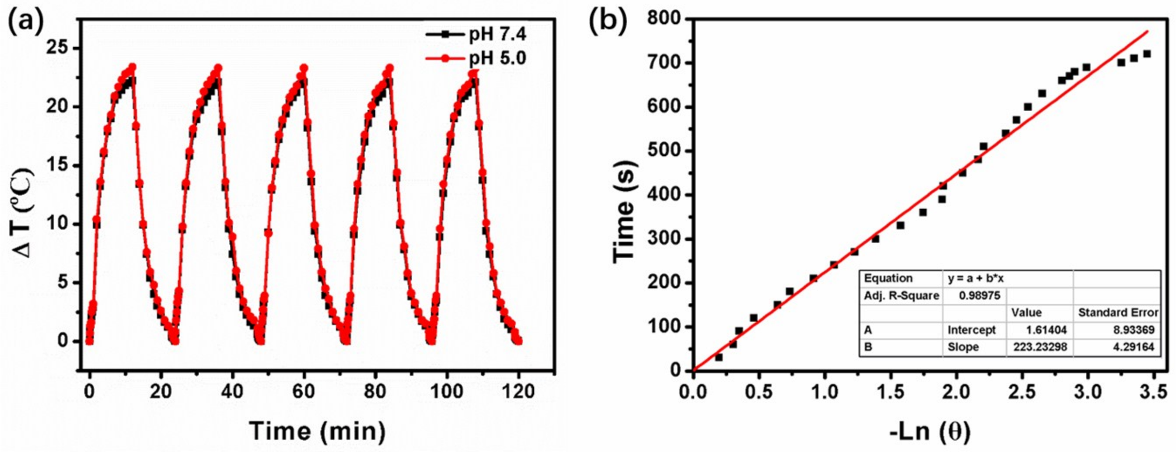

Figure S2 (a) Photothermal stability test of DPP-NF NPs solution under photothermal ramp-up and natural cooling cycles at different pH values. (b) Time constant for heat transfer from the system is determined to be τ_s = 223.23 s by applying linear time data from cooling period (after 720 s) *versus* negative natural logarithm of driving force temperature obtained from the cooling stage of Figure 2(d).

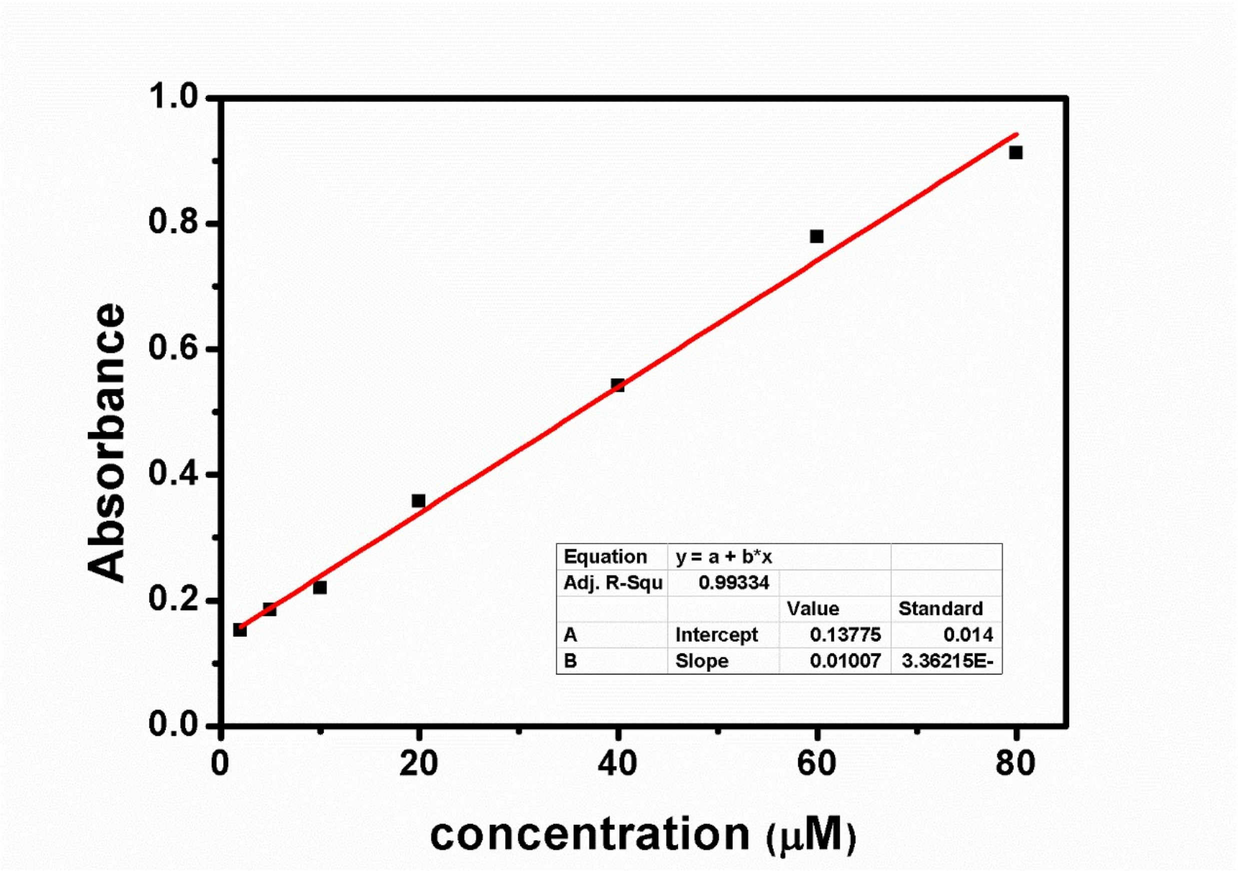

Figure S3 Standard curve of $NaNO_2$.

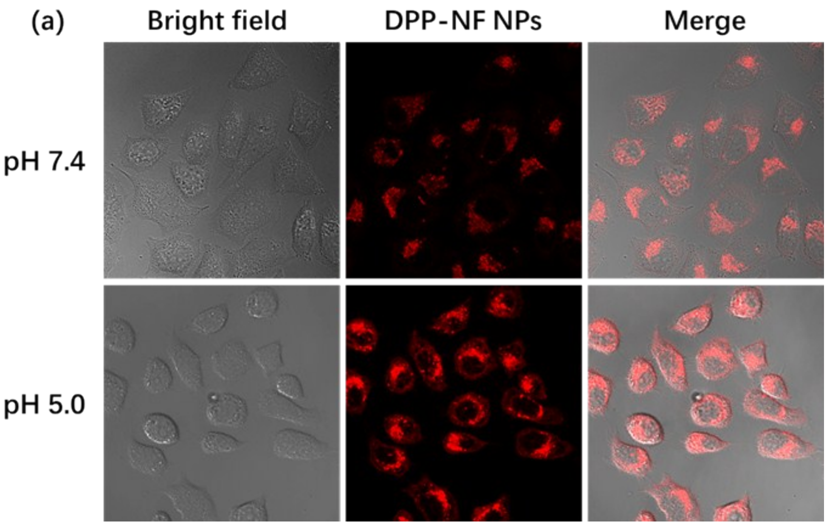

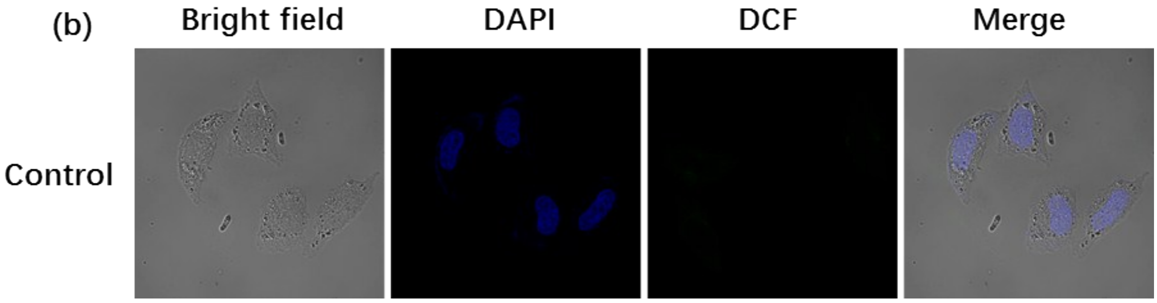

Figure S4. (a) Confocal images of Hela cells incubated with DPP-NF NPs at pH 7.4 and 5.0. (b) Confocal images of DAPI and DCFH-DA stained HeLa cells pre-incubated with DPP-NF NPs without irradiation.

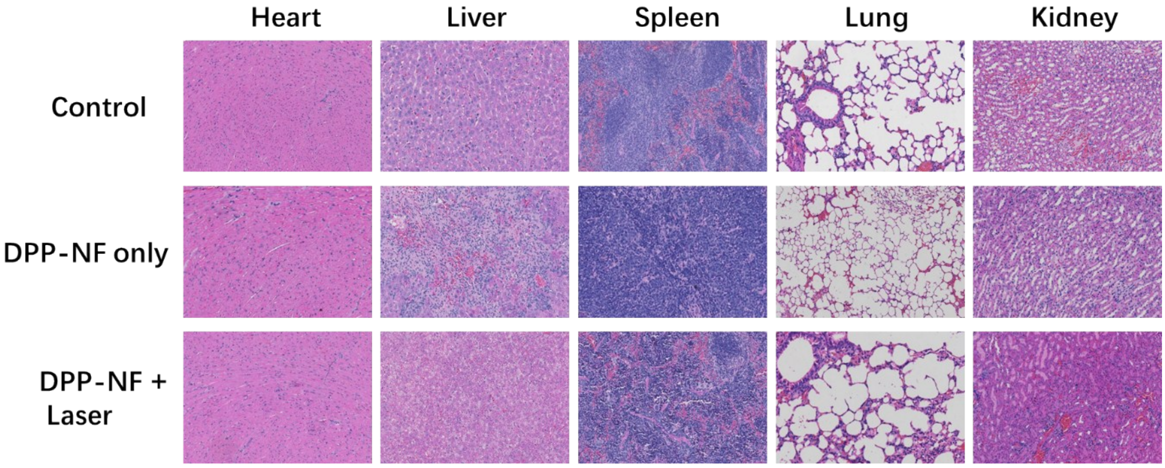

Figure S5 H&E stained images of major organs (heart, liver, spleen, lung, and kidney) for different groups after 26 days treatment.
